# Author Correction: T cell responses to SARS-CoV-2 spike cross-recognize Omicron

**DOI:** 10.1038/s41586-022-04708-y

**Published:** 2022-04-08

**Authors:** Roanne Keeton, Marius B. Tincho, Amkele Ngomti, Richard Baguma, Ntombi Benede, Akiko Suzuki, Khadija Khan, Sandile Cele, Mallory Bernstein, Farina Karim, Sharon V. Madzorera, Thandeka Moyo-Gwete, Mathilda Mennen, Sango Skelem, Marguerite Adriaanse, Daniel Mutithu, Olukayode Aremu, Cari Stek, Elsa du Bruyn, Mieke A. Van Der Mescht, Zelda de Beer, Talita R. de Villiers, Annie Bodenstein, Gretha van den Berg, Adriano Mendes, Amy Strydom, Marietjie Venter, Jennifer Giandhari, Yeshnee Naidoo, Sureshnee Pillay, Houriiyah Tegally, Alba Grifoni, Daniela Weiskopf, Alessandro Sette, Robert J. Wilkinson, Tulio de Oliveira, Linda-Gail Bekker, Glenda Gray, Veronica Ueckermann, Theresa Rossouw, Michael T. Boswell, Jinal N. Bhiman, Penny L. Moore, Alex Sigal, Ntobeko A. B. Ntusi, Wendy A. Burgers, Catherine Riou

**Affiliations:** 1 Institute of Infectious Disease and Molecular Medicine, University of Cape Town, Observatory, Cape Town, South Africa; 2 Division of Medical Virology, Department of Pathology, University of Cape Town; Observatory, Cape Town, South Africa; 3 Africa Health Research Institute, Durban, South Africa; 4 School of Laboratory Medicine and Medical Sciences, University of KwaZulu-Natal, Durban, South Africa; 5 National Institute for Communicable Diseases of the National Health Laboratory Service, Johannesburg, South Africa; 6 SA MRC Antibody Immunity Research Unit, School of Pathology, Faculty of Health Sciences, University of the Witwatersrand, Johannesburg, South Africa; 7 Department of Medicine, University of Cape Town and Groote Schuur Hospital; Observatory, Cape Town, South Africa; 8 Department of Immunology, University of Pretoria, Pretoria, South Africa; 9 Tshwane District Hospital, Tshwane, South Africa; 10 Centre for Viral Zoonoses, Department of Medical Virology, University of Pretoria, Pretoria, South Africa; 11 KwaZulu-Natal Research Innovation and Sequencing Platform, University of KwaZulu-Natal, Durban, South Africa; 12 Center for Infectious Disease and Vaccine Research, La Jolla Institute for Immunology, La Jolla, CA USA; 13 Department of Medicine, Division of Infectious Diseases and Global Public Health, University of California, San Diego (UCSD), La Jolla, CA USA; 14 Wellcome Centre for Infectious Diseases Research in Africa, University of Cape Town, Observatory, Cape Town, South Africa; 15 Department of Infectious Diseases, Imperial College London, London, UK; 16 The Francis Crick Institute, London, UK; 17 Centre for Epidemic Response and Innovation, Stellenbosch University, Stellenbosch, South Africa; 18 Desmond Tutu HIV Centre, University of Cape Town, Cape Town, South Africa; 19 South African Medical Research Council, Cape Town, South Africa; 20 Department of Internal Medicine, University of Pretoria and Steve Biko Academic Hospital, Pretoria, South Africa; 21 Centre for the AIDS Programme of Research in South Africa, Durban, South Africa; 22 Max Planck Institute for Infection Biology, Berlin, Germany; 23 Cape Heart Institute, Faculty of Health Sciences, University of Cape Town; Observatory, Cape Town, South Africa

**Keywords:** Viral infection, Lymphocyte activation, SARS-CoV-2

Correction to: *Nature* 10.1038/s41586-022-04460-3 Published online 31 January 2022

In this Article, the name of author Jinal N. Bhiman was incorrectly shown as ‘Jinal Bihman’. The original Article has been corrected online

